# Changes in Visual Performance under the Effects of Moderate–High Alcohol Consumption: The Influence of Biological Sex

**DOI:** 10.3390/ijerph18136790

**Published:** 2021-06-24

**Authors:** Miriam Casares-López, José J. Castro-Torres, Sonia Ortiz-Peregrina, Francesco Martino, Carolina Ortiz

**Affiliations:** Laboratory of Vision Sciences and Applications, Department of Optics, Facultad de Ciencias (Edificio Mecenas), University of Granada, Avenida Fuentenueva s/n, 18071 Granada, Spain; soniaortiz@ugr.es (S.O.-P.); francesco@correo.ugr.es (F.M.); ortizh@ugr.es (C.O.)

**Keywords:** alcohol consumption, visual deterioration, biological sex, retinal image quality, visual discrimination capacity, stereopsis

## Abstract

The purpose of this study was to analyze the changes in visual functions under the effects of moderate–high breath alcohol concentrations (BrACs), and the influence of biological sex on visual deterioration, considering different factors. A total of 37 healthy habitual alcohol users were enrolled in the experiment. The participants underwent a baseline session and a second session after an intake of 450 mL of red wine, so that all of them reached a BrAC above 0.25 mg/L. Visual performance was assessed by measuring the contrast sensitivity function, the halo perception, the stereopsis, and finally the retinal image quality. A Visual Deterioration Score (VDS) was calculated using the deterioration of these visual variables. All visual functions analyzed were significantly impaired following alcohol consumption (*p* < 0.05). The VDS was associated with the BrAC (ρ = −0.476). The VDS was also significantly higher in females, with the BrAC having a significant effect on the variability of the VDS in males and females (*p* < 0.05). However, the body mass index showed no significant effect (*p* > 0.05). Visual functions were significantly impaired under the influence of alcohol, and this deterioration was greater in females. The deterioration depends on the BrAC reached, being the primary thing responsible for the differences observed between males and females.

## 1. Introduction

Alcohol abuse is an important public health concern; in 2016, it was responsible for 3 million deaths worldwide and 132.6 million disability-adjusted life years. Moreover, alcohol-related mortality is higher than that from certain diseases, such as HIV, diabetes, and tuberculosis [1]. According to the literature, the majority of visual functions are altered by alcohol, although not on an equal basis. The contrast sensitivity function is impaired by alcohol consumption [2,3,4], although it is not clear if this deterioration depends on the spatial frequency. One ambiguous point is whether alcohol consumption actually affects stereopsis, since few studies have investigated this aspect. Some authors have found a clear deterioration of near stereoacuity following alcohol consumption [3], but others have observed no effect [5,6]. What is clear is that alcohol does affect the accommodative function and vergence system [7,8,9], which could influence stereoacuity. In this sense, some works have shown that exophoria (increased at near vision tasks under the effects of alcohol) has a lesser effect on stereopsis, although it may vary depending on the magnitude of the exophoria [10,11]. However, esophoria (increased at distance) has a greater influence [8,12,13]. The effects of alcohol consumption on other visual functions, such as halo perception (and other night vision dysphotopsia) and objective measurements (such as the retinal image quality) have been less studied. Studies have reported a deterioration of these two visual functions under the effects of different doses of alcohol [14,15], although the retinal image quality was only studied for a fixed pupil of 4 mm. It is important to consider these changes in visual function, since they could have an impact in highly visual tasks, such as driving, as some authors have recently reported [2].

Biological sex is one of the main factors determining the effects of alcohol (and one of the most widely studied). It is known that, due to sex differences in alcohol metabolism, women are generally more vulnerable to the toxic effects of ethanol, and reach higher BrAC levels than men when the same amount of alcohol is consumed [16,17,18]. On the other hand, some authors have observed differences between males and females when performing certain visuo-perceptual tasks [19]. Many authors have discussed the relationship between visual deterioration and blood alcohol concentration (BAC, related to BrAC by a ratio of 2:1, a BAC of 0.50 g/L (0.05%) equals a BrAC of 0.25 mg/L). The majority have observed that changes in vision mainly occurred in cases of moderate and high alcohol doses, usually involving BAC levels above 0.05% [3,8,14,20,21]. Some authors have reported that higher alcohol concentrations (either BAC or BrAC) are associated with greater visual impairment following alcohol consumption [5,14]. In contrast, other authors have found no such association [5,22], and it seems that whether an association is recorded or not may depend on the visual function analyzed. However, results from previous studies on the influence of alcohol on psychomotor and cognitive functions indicate that several factors should be considered to determine whether alcohol intake will produce a negative effect, as well as the intensity of this effect. Furthermore, some authors have reported that females have a shorter non-invasive break-up time (NIBUT) i.e., a poor tear film stability [23], which may have an impact on visual quality. On the other hand, body mass index (BMI) could also have an influence in the possible effects of alcohol on visual performance, since some authors have reported that a higher BMI is associated with a lower volume distribution of alcohol in males and females [24]. Considering this, it is reasonable to hypothesize that females could experience different visual impairment than males, but very little has been published on the role biological sex plays in the visual deterioration caused by alcohol use, and the results are inconclusive [14].

The aim of this work was, therefore, to study different aspects of visual function following alcohol use, with moderate–high BrAC levels being reached after the subjects consumed a controlled dose of alcohol, including an analysis of whether biological sex is associated with the visual deterioration produced after alcohol intake, considering the BrAC level and the BMI of the participants.

## 2. Materials and Methods

### 2.1. Subjects

A total of 37 participants were included in the study, 20 females and 17 males (according to their biological sex, which, in all cases, matched the assumed gender), aged from 20 to 56 (mean age of 30.4 ± 2.5 and 26.2 ± 1.7 years in males and females, respectively). All participants signed an informed consent form according to the declaration of Helsinki, and the study was approved by the Human Research Ethics Committee of the University of Granada (921/CEIH/2019). As inclusion criteria, it was established that all participants should have near and distance visual acuity (VA), with the best optical correction of 1.0 (decimal notation), have no pathological disease or condition that could limit binocular visual performance, have never undergone refractive surgery, were not taking any medication that could affect their vision or interfere with the alcohol absorption process, and be social drinkers. Before the experimental sessions, the state of the binocular vision was tested by measuring the near and far horizontal phoria. Of the initial 39 participants, one was discarded due to abnormal results, and another was excluded due to simulator-sickness. Height and weight were measured for each participant, and the body mass index (BMI) was calculated. To ensure that none of them had any alcohol dependence or alcoholic tendency, they took the Alcohol Use Disorders Identification Test (AUDIT). This is a 10-item questionnaire to assess alcohol use in the past year, and enables the detection of harmful alcohol-related behaviors. Each answer is scored from 0 to 4, with 0 indicating no use of alcohol. More information on this test can be found elsewhere [25]. All participants scored a total of 8 or less, meaning that none had an alcohol problem.

### 2.2. Alcohol Administration

Each participant underwent two randomized experimental sessions, two weeks apart, and at the same time of the day: one in normal conditions (baseline), and the other after consuming alcohol (aAC). Prior to starting the baseline session, the subjects received training for some visual tasks to ensure that they understood how to perform them properly. To simulate social drinking and create a realistic drinking atmosphere, the alcohol used was red wine with an alcohol content of 13.5% (Bodegas Pago de Almaraes, S.L. Benalúa de Guadix, Granada, Spain) [14,15]. Each participant was provided with 450 mL of wine, in such a way that we obtained different alcohol rates. This amount was selected based on previous experiments carried out in our laboratories [2,9]. For this amount of wine, all participants reached or exceeded the legal limit of alcohol for driving in Spain, and most other countries (a BrAC of 0.25 mg/L, equivalent to a BAC of 0.05%) [1]. The Widmark equation was used to ensure that, for 450 mL of wine, all participants reached a BAC level higher than 0.05% according to their weight:

$$\mathrm{BAC}\left(g/L\right)=\frac{\mathrm{amount}\;\mathrm{of}\;\mathrm{ingested}\;\mathrm{alcohol}\;\left(g\right)}{\mathrm{weight}\;\left(\mathrm{kg}\right)\times \mathrm{distribution}\;\mathrm{factor}}$$

According to previous works, the distribution factor was assumed as 0.60 for females and 0.70 for males [26].

The same visual tasks and ocular measurements were randomly performed in both experimental sessions (baseline and aAC). Alcohol ingestion took place in our laboratories within a 30–40 min period and two hours after the last meal. Thirty minutes after consuming alcohol, when it had time to be absorbed, breath alcohol content (BrAC) was measured for the first time, using the breath analyzer Dräger Alcotest 6820 (Dräger Safety AG & Co. Lubeck, Germany), which provides the BrAC level expressed in milligrams of ethanol per liter of exhaled air (mg/L). Every 20 min, a new measurement was made to ensure that the BrAC level remained stable, with four different measurements recorded by the end of the aAC session. The four measurements were averaged to obtain the mean BrAC value for each participant.

### 2.3. Vision Assessment

Different visual parameters were assessed in this study: contrast sensitivity, near and distance stereopsis, visual discrimination capacity under low-illumination conditions (halo perception), forward retinal scattering, and retinal image quality. The subjects performed the vision tests with best correction. For the monocular measurements, one eye was randomly selected for each participant [27]. The mean values and the deterioration of all the visual variables were provided. The deterioration was calculated as the difference between the baseline and aAC values.

#### 2.3.1. Contrast Sensitivity and Night Vision

Contrast sensitivity was assessed in monocular and binocular conditions using the Pola VistaVision monitor (DMD MedTech, Villarbasse, Torino, Italy). The screen implements a contrast scale of 13 values, stablished according to a logarithmic scale (log SC). This test comprises sinusoidal grids, with positions that could be right, left, or vertical, such that observers should indicate the direction of inclination. Eight different contrast levels were assessed in this test, calculated according to the Michelson contrast modulation formula, at six spatial frequencies: 0.75, 1.5, 3, 6, 12, and 18 cycles per degree (cpd). The test was performed at 3 m in dim surroundings.

Night vision was assessed by monocularly testing the participants’ visual discrimination capacities under low illumination conditions. Visual discrimination capacity was tested using a halometer, the Halo v1.0 (University of Granada, Granada, Spain), freeware software available in the institutional repository (http://hdl.handle.net/10481/5478 access on: 14 April 2021). This test quantifies the visual disturbances perceived by the observer under the influence of a high-luminance stimulus in dim surroundings [28]. At a distance of 2.5 m, each subject had to detect peripheral stimuli (with a luminance of 61 cd/m^2^) around the central high-luminance stimulus (176 cd/m^2^). At the end of the test, the visual disturbance index (VDI) was provided. VDI results range from 0 to 1, in such a way that the higher the VDI, the greater the disturbance and the halo perceived [15,28]. Under the same viewing conditions, pupil size was measured using a Colvard pupilometer (OASIS Medical, Inc., Glendora, CA, USA).

#### 2.3.2. Stereopsis

Stereopsis was evaluated by means of near and distance stereoacuity under photopic conditions. Near stereoacuity was tested using the Frisby Near Stereotest, which has been proven reliable for assessing stereoscopic vision [29]. The test consists of three plates, each with a different thickness (6, 3, and 1.5 mm) and four squared random-patterns printed on the plate. One of the four patterns holds a circular target patch of elements at a depth relative to the surroundings, in such a way that participants have to recognize in which of the four random patterns the circular patch is located. According to the thickness of the plate, and the distance at which the plate is positioned with respect to the observer, different stereopsis levels can be assessed, ranging from 600 to 5 arc sec.

Distance stereoacuity was tested at 5.5 m using the differentiated stereo D8 polarized test implemented using the Pola VistaVision monitor (DMD Med Tech Srl., Torino, Italy), following the recommendations of the Wertheimer for good stereopsis assessment [30]. The stereo test evaluates eight disparities from 300 to 10 arc sec (300, 240, 180, 120, 60, 30, 20, and 10 arc sec) using polarized vertical lines. For each stereoacuity test, five vertical lines were displayed simultaneously along a row on the monitor (two rows or stereoacuities were tested per screen displayed), one of which showed disparity that had to be perceived stereoscopically. For this test, the observer wore polarized glasses provided by the monitor manufacturer, and the task was to recognize the vertical line perceived stereoscopically.

#### 2.3.3. Retinal Image Quality

The retinal image quality was objectively tested using the double-pass commercial device OQAS II (Optical Quality Analysis System. Visiometrics S.L., Terrassa, Spain), which enables only monocular measurements. The OQAS II provides the retinal double-pass image of a point source of light using infrared light (780 nm) coupled to an optical fiber. With this system, the light from a point source (infrared diode laser) is collimated and then enters the eye, forming an image in the retina (first pass); then, the reflected light from the retina is collected by a lens, forming an image in a CCD sensor (second pass) [31]. The device gives information on ocular aberrations and scattering, which are responsible for the deterioration of the optical quality of the eye [32]. For that, the Strehl ratio, the MTF cut-off frequency, and the objective scatter index (OSI) were measured. The Strehl ratio is defined as the ratio between the 2D-Modulation Transfer Function area (2D-MTF) of the eye and the diffraction-limited 2D-MTF area, where the MTF curve represents how the eye reproduces contrast as a function of spatial frequency. The values of the Strehl ratio range from 0 to 1, with low values for this parameter, indicating a poorer retinal image quality [14]. The MTF cut-off is the frequency at which the MTF reaches a value of 0.01 [33]. Likewise, the lower the MTF cut-off frequency, the poorer the retinal image quality. On the other hand, considering the retinal double-pass image of a point source of light, the OSI is calculated as the ratio between the light intensity within an annular area of 12 and 20 arc min and the intensity of the central peak [31,34]. Typical OSI values in normal eyes are lower than 1.0, indicating low amounts of scatter and, thus, a good-quality retinal image [31]. The MTF cut-off and Strehl ratio were measured for two fixed pupil diameters: 4 and 5 mm. The OSI was measured for a pupil diameter of 4 mm. The test was performed in dim surroundings, and we ensured that all the participants had a pupil size of 5 mm or wider under these lighting conditions.

### 2.4. Data Analysis

To statistically analyze the results, we used the software SPSS statistics (SPSS Inc., Chicago, IL, USA). The normality of the data was tested with a Kolmogorov–Smirnov test. Kruskal–Wallis and Mann–Whitney U tests were performed to analyze the influence of the spatial frequency and viewing conditions, respectively, on contrast sensitivity deterioration, providing the degrees of freedom, the statistic (chi-square c2 and Z respectively), and the *p*-value. Differences between the baseline and aAC results for normal data were analyzed with a *t*-test for related samples. For non-normal data, the differences were analyzed using a Wilcoxon test, providing the degrees of freedom, the statistic (t and Z respectively), and the *p*-value.

To analyze any sex differences in the visual deterioration of normal variables, a *t*-test for independent samples was run, using biological sex as a factor and providing the degrees of freedom, the t statistic, the *p*-value, and Cohen’s d. In the case of non-normal data, a Mann–Whitney U test was used.

A visual deterioration score (VDS) was calculated using the deterioration results of each variable. For this, the z-scores corresponding to the deterioration of each visual variable were calculated and then averaged, with the same weight being assigned to all variables. The deterioration of some visual variables (the CS, the Strehl ratio, and the MTF cut-off) was multiplied by −1. In this way, a VDS was obtained for each participant, in such a way that the more negative the VDS, the greater the deterioration under the effects of alcohol. The same method has been used before by some authors to calculate an overall score to assess driving performance [2,35]. To compare the VDS and the BrAC in males and females, a *t*-test for independent samples was run, using biological sex as a factor. A Spearman correlation test was performed to analyze the association between the BrAC level and the VDS, and between the non-normal visual variables. An ANCOVA test was used to analyze the impact of biological sex on visual deterioration, including the BrAC, the BMI, and the age as covariates, since these factors could influence the observed effects of alcohol [24,36,37]. The degrees of freedom, the F statistic, the *p*-value, and the effect size (h2) were provided. A significance level of 95% was considered for all the tests.

## 3. Results

The mean BrAC reached by the participants (37 in total) after consuming 450 mL of red wine was 0.34 ± 0.01 mg/L (equivalent to a BAC level of 0.07%), with a mean BrAC of 0.30 ± 0.02 mg/L for males and 0.38 ± 0.02 mg/L for females. All the participants exceeded the legal alcohol limit for driving in most countries (0.25 mg/L).

### 3.1. Visual Discrimination Capacity and Pupil Size

**Figure 1:**
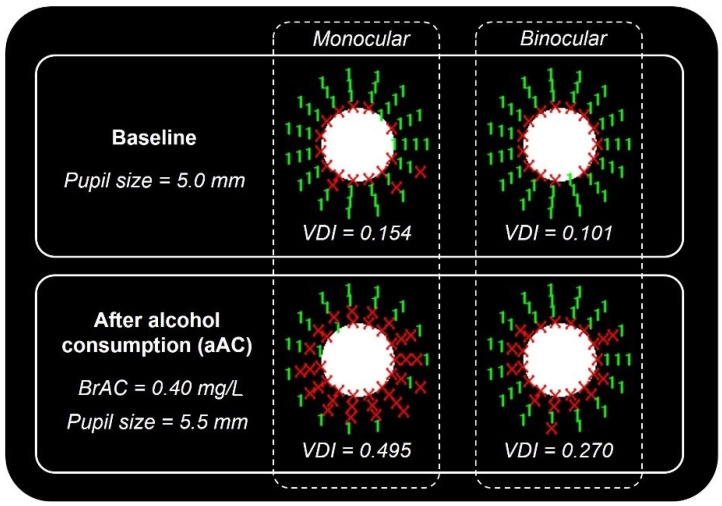
Graphic monocular and binocular results for the VDI and pupil size of one of the participants in both experimental conditions (baseline and aAC). The red symbols (X) correspond to peripheral stimuli not detected by the subject, revealing the shape of the halo.

Table 1 shows the mean visual disturbance index (VDI) along with pupil size, as well as the deterioration observed in the aAC condition. The deterioration of these variables (VDI and pupil size) following alcohol consumption was negative, since this was calculated as the difference between the results from the baseline and aAC sessions, and higher VDI values indicated a greater perception of halos. Both monocular and binocular VDI were significantly higher after alcohol consumption, indicating that halo perception increased. Pupil size was also significantly higher under these conditions (Table 1). An example of the VDI deterioration is shown in Figure 1, which presents the graphic results of the visual discrimination capacity: the number 1 and the symbol “X” refer to a detected and non-detected peripheral stimulus, respectively. As Figure 1 demonstrates, the number of non-detected stimuli peripheral to the central one is higher after alcohol use, especially under monocular viewing.

### 3.2. Stereopsis

The near and distance stereopsis results and the corresponding deterioration can be seen in Table 1. It should be noted that, in the two experimental conditions, distance stereopsis was worse than near stereopsis. The deterioration of this visual function was also negative, as higher values indicate poorer stereopsis. Near stereopsis was significantly impaired under the effects of alcohol, as was distance stereopsis. The calculated quotient (baseline/aAC) of distance and near stereopsis was 0.48 ± 0.30 and 0.58 ± 0.28, respectively. This indicates a greater impairment of the distance stereopsis, but not significant (Z(72) = 1.358; *p* = 0.174; Cohen’s d = 0.320), with the mean stereoacuity in the aAC condition being far above the normal stereopsis values (around 40 arc sec, although this may change slightly depending on the test used) [38].

### 3.3. Contrast Sensitivity

Contrast sensitivity (CS) also deteriorated after alcohol intake (Table 1). This deterioration was a positive value (higher CS values indicate better visual function). According to the Mann–Whitney test results, there were significant differences between monocular and binocular CS deterioration ((35) = −3.344; *p* = 0.001; Cohen’s d = 0.844), this being higher in monocular conditions. Likewise, the Kruskal–Wallis analysis revealed that the contrast sensitivity deterioration differed significantly for the spatial frequencies analyzed (Z(5) = 20.881; *p* = 0.001; Cohen’s d = 0.398). The Wilcoxon test result showed that monocular CS deterioration was significant for the spatial frequencies of 0.75 cpd (Z(36) = −4.041; *p* < 0.001; Cohen’s d = 1.778), 1.5 cpd (Z(36) = −3.819; *p* < 0.001; Cohen’s d = 1.613), 3 cpd (Z(36) = −2.918; *p* = 0.004; Cohen’s d = 0.703), 6 cpd (Z(36) = −3.457; *p* = 0.001; Cohen’s d = 1.381), and 18 cpd (Z(36) = −3.132; *p* = 0.002; Cohen’s d = 1.201). Binocular contrast sensitivity was also significantly deteriorated for all spatial frequencies: 0.75 cpd (Z(36) = −3.494; *p* < 0.001; Cohen’s d = 1.403), 1.5 cpd (Z(36) = −2.449; *p* = 0.014; Cohen’s d = 0.880), 3cpd (Z(36) = −2.530; *p* = 0.011; Cohen’s d = 0.915), 6 cpd (Z(36) = −3.457; *p* = 0.001; Cohen’s d = 1.381), 12 cpd (Z(36) = −3.288; *p* = 0.001; Cohen’s d = 1.285) y 18 cpd (Z(36) = −3.094; *p* = 0.002; Cohen’s d = 1.182). In addition, we observed that a higher VDI was associated with a lower monocular CS (ρ = −0.461; *p* < 0.001), but also with a lower binocular CS (ρ = −0.558; *p* < 0.001), indicating that participants with better contrast sensitivity reported better visual discrimination.

### 3.4. Retinal Image Quality

The retinal image quality results are shown in Table 2. As can be observed, retinal image quality was negatively affected by alcohol intake. For the Strehl ratio and MTF cut-off, the higher the values, the better the image quality and, therefore, the deterioration is positive. On the other hand, higher OSI values indicate greater intraocular scattering and, consequently, a worse image quality, with the deterioration being negative. The MTF cut-off was significantly lower under the effect of alcohol for both artificial pupil sizes (4 mm and 5 mm), with this deterioration being higher, but not significantly so (*p* > 0.05), in the case of the 5 mm pupil. The deterioration of the Strehl ratio was also statistically significant and equal for the two pupil sizes. The OSI was significantly higher following alcohol consumption, indicating significantly increased intraocular scattering under these conditions. We also observed that a higher OSI was associated with a higher VDI (ρ = 0.288; *p* = 0.042). 

### 3.5. Influence of Biological Sex

**Figure 2:**
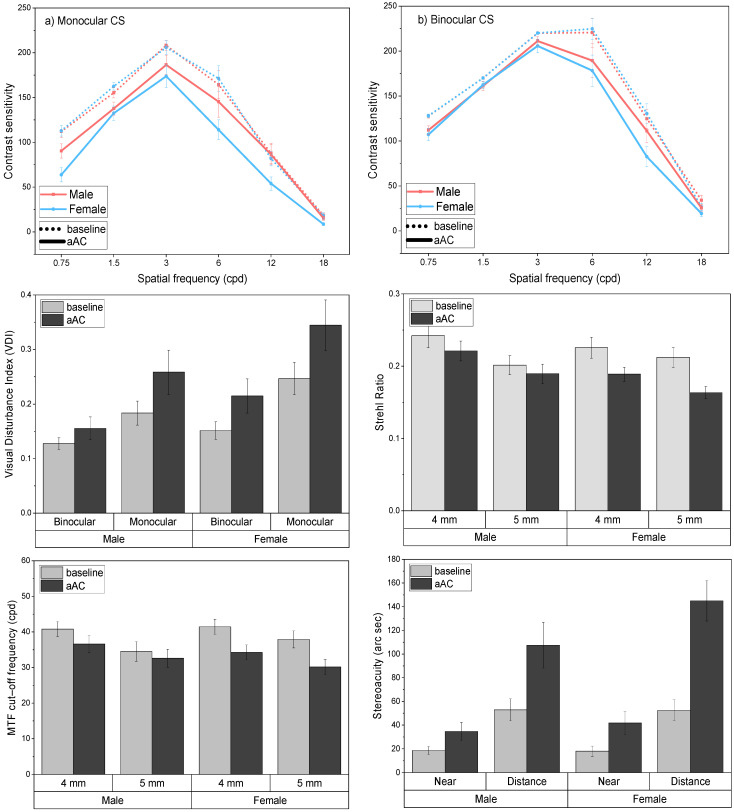
Mean contrast sensitivity function, visual deterioration index (VDI), stereopsis (near and distance stereoacuity), and retinal image quality (MTF cut-off and Strehl Ratio) in males and females. Standard errors included.

The Mann–Whitney U test revealed that females reached a higher BrAC than males (*p* < 0.05) and had a lower BMI (*p* < 0.05), but no significant age differences were observed (*p* > 0.05). The mean results of the visual variables for males and females are presented in Figure 2. As can be observed, the deterioration under the effects of alcohol was greater in females for all visual variables. The results of the *t*-test and Mann–Whitney U test (Table 3) confirmed that all visual functions were more impaired in females, particularly the monocular CS, the Strehl ratio, and the MTF cut-off for the fixed pupil of 5 mm, with biological sex having a significant effect on the deterioration of these variables.

**Figure 3:**
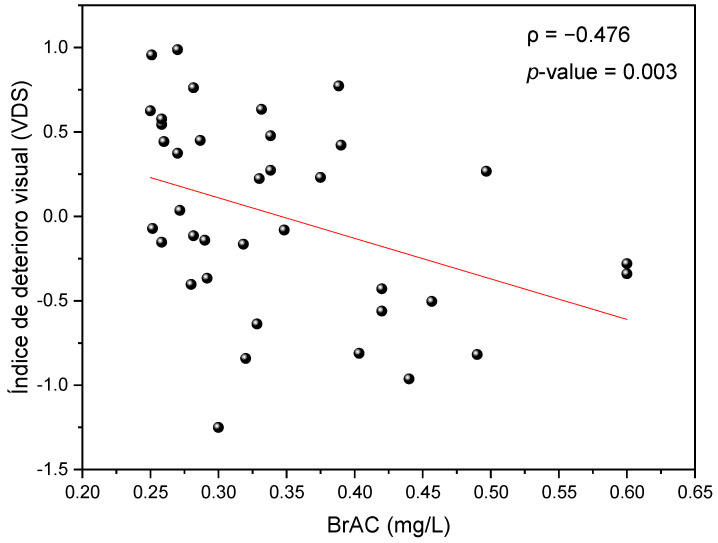
Visual Deterioration Score (VDS) as a function of the breath alcohol content (BrAC, in mg/L) reached for each participant.

The mean VDS was −0.22 ± 0.12 for females, and 0.27 ± 0.12 for males; the VDS in women is negative, indicating a greater visual deterioration. A *t*-test for independent samples was performed to analyze the effect of biological sex on the VDS, and significant differences were observed (t(36) = 2.992; *p* = 0.005; Cohen’s d = 0.893). The Spearman test showed a significant descending correlation between the VDS and BrAC level (ρ = −0.467; *p* = 0.004). This association indicates that for higher alcohol levels, visual deterioration was also greater, since the more negative the VDS value, the greater the visual impairment induced by alcohol intake (Figure 3). Because the BrAC level correlated with the VDS, an ANCOVA test was performed, with VDS selected as the dependent variable, biological sex as a fixed factor (independent variable), and with BrAC and age as covariates.

According to the ANCOVA results (Table 4), a significant effect of the BrAC (*p* = 0.041) was observed when controlling for BrAC, the BMI and age, in such a way that the sex differences in the VDS were no longer significant. However, the BMI and the age had no significant effect. This means that the sex differences observed in the *t*-test for the VDS were mainly due to differences in BrAC, rather than in BMI.

## 4. Discussion

Our results showed a significant deterioration in all the visual functions analyzed when the BrAC level was above 0.25 mg/L, the legal limit for driving in most countries. Both monocular and binocular visual discrimination capacity in dim lighting conditions, characterized by means of the visual disturbance index (VDI), were impaired after alcohol consumption, with an increase in halo perception and other visual disturbances. Pupil size measured under the same viewing conditions was also wider under the effects of alcohol. Other authors have also reported deteriorated night vision under the effects of alcohol. Specifically, there is a deterioration in the VDI, as well as an increased pupil size, particularly for high BrAC levels [14,15,39]. The increased pupil size observed in this study may be partially responsible for the changes observed in the visual discrimination capacity, since it increases the amount of intraocular scattering and decreases retinal image quality [14,40]. However, the deterioration of night vision is not only due to pupil size changes, but also to the effects alcohol has on the tear film [14]. This seems feasible as, after consuming alcohol, this substance has actually been found in tears, and increased osmolarity of the tears has also been observed, leading to faster tear film evaporation and a decreased break-up time [41,42].

According to our findings, the contrast sensitivity (CS) was also deteriorated following alcohol consumption, in both monocular and binocular viewing conditions. These results are in line with previous findings, as most authors have reported decreased contrast sensitivity under the effects of alcohol for moderate–high alcohol doses [43,44]. Although we found that CS was impaired for each spatial frequency, this deterioration was not equal for all of them, in agreement with the findings of other authors [3,4,22]. However, not all studies agree on which spatial frequencies are most affected, as this may depend on the alcohol concentration reached, the sensitivity of the test, the characteristics of the sample, and other experimental conditions. Andre et al. [45] reported that all spatial frequencies were equally altered for a stationary target, but their sample was smaller and included only young subjects, while our study encompassed older participants, with more reduced contrast sensitivity for high spatial frequencies [46].

In this work, we observed that retinal image quality was degraded under the effects of alcohol. This deterioration was evidenced as a decreased MTF cut-off frequency and a lower Strehl ratio for the two artificial pupil sizes (4 and 5 mm), indicating an increase in both intraocular scattering and aberrations. Similar results for a 4 mm fixed pupil were reported by other authors [14,15], although the amount of alcohol provided in those experiments was not controlled for each participant, and various breath alcohol concentrations (BrACs) were obtained. Our results confirmed that this deterioration was also present for a 5 mm fixed pupil, which was, in fact, closer to the real pupil size under these experimental conditions (Table 1). It is known that image quality is pupil-size dependent [47,48], in such a way that the larger the pupil, the greater the intraocular scattering and the more ocular aberrations. This would explain the increased deterioration observed for the 5 mm-pupil MFT cut-off compared to that for the 4 mm-pupil (Table 2). However, the deterioration in the Strehl ratio after alcohol consumption was equal for both 4 and 5 mm (Table 3). The objective scatter index (OSI), which provides information on intraocular scattering, was higher following alcohol consumption, in line with previous research in which the amount of alcohol consumed varied among the participants [14]. Our results corroborate the tendency towards image quality deterioration after alcohol intake, since all the participants in our study ingested the same amount of red wine (450 mL). Increased intraocular aberrations and more scattered light both negatively affect the point spread function (PSF) in the retina, which translates into a deteriorated retinal image quality. As the double-pass measurements and the retinal image quality comparisons between the two experimental conditions (baseline and aAC) were performed using a fixed artificial pupil of 4 or 5 mm, it is expected that, in this case, pupil size was not entirely responsible for the deterioration in retinal image quality. However, the effect of alcohol on the tear film has been suggested as another possible cause of deteriorated retinal image quality [14], since alcohol has been shown to exert a negative effect on this, as discussed above [41,42]. Some authors have reported that the OSI value is a function of retinal straylight [49]. In line with this, we observed that higher OSI values and, therefore, an increased influence of retinal straylight, are associated with a greater perception of halos (VDI).

Contrary to the other visual functions analyzed in this work, and considering published studies, there is no agreement as to whether alcohol consumption affects stereopsis, although some authors have found that this deteriorates under the effect of other drugs, such as cannabis [50,51]. According to our results, it is clear that both near and distance stereopsis are highly impaired under the effects of alcohol. However, the mean near stereoacuity achieved in the aAC condition is still within normal values (40 arc sec). Watten and Lie [3] also reported this conundrum. Although other authors found that alcohol had no effect on stereoacuity [5,6,52], Watten and Lie reported that it was strongly impaired following alcohol consumption. All of these experiments involved a high blood alcohol concentration (BAC) similar to our BrAC results. However, just as we did in this study, Watten and Lie analyzed a larger sample and this may be a reason behind this discordance. Furthermore, the stereoscopic tests used in our study were more sensitive than the stereoscopic tests used by the majority of authors, who used the TITMUS test. This only allows stereopsis to be assessed up to 40 arc sec, which is higher than the mean stereoacuity achieved by our participants in the aAC condition. Although Nawrot et al. [52] used a random dot test that allowed stereopsis to be assessed up to 20 arc sec, this was still less sensitive than our two stereotests. In fact, it seems reasonable that stereopsis deteriorates following alcohol consumption, considering that alcohol negatively affects the ocular motor system, which is responsible for vergence [6]. In this sense, some authors have observed increased esophoria at distance under the effects of alcohol [7,8]. Saladin showed that esophoria affected stereoacuity [10], which would explain the deteriorated stereopsis. However, other authors have reported that depth perception is affected because of the effect alcohol has on the slow eye movements that control motion parallax, but not stereoacuity [52]. For this reason, further investigation is needed to clarify these aspects. Our results also showed that stereopsis was more impaired at distance, which is in line with previous findings indicating that heterophoria also increases at distance [7,8].

These results should be considered, since this impairment could impact the performance of daily activities that require fine and accurate vision, such as driving [53,54]. In fact, it has been observed that alcohol intake impairs driving performance [55,56], partially due to the visual deterioration of some visual functions [2].

Our results show that the impairment resulting from alcohol consumption was greater in females for all visual variables, especially in the case of the monocular CS, and retinal image quality (Strehl ratio and MTF cut-off) for a 5 mm pupil. From previous studies, it is not clear whether visual deterioration is different in males and females. Castro et al. found that the deterioration of the visual discrimination capacity was greater in females, but no difference was observed in the deterioration of retinal image quality between females and males [14]. However, the amount of alcohol consumed differed between the participants. In this sense, Niaura et al. reported few biological sex-related differences in the effects of alcohol consumption on psychomotor skills [57]. As we have reported, it seems that the visual deterioration recorded in males and females may be different according to the visual function analyzed. Considering this, the calculation of the visual deterioration score (VDS), which includes all the visual variables analyzed in this study, provides an overview of how visual function changes following alcohol consumption for each participant. In addition, the VDS helps us to understand how vision changes as a function of different factors, like biological sex in this case. The *t*-test showed that the VDS was higher in females, and we also observed that females reached higher BrAC levels than males, which was not surprising, as it is clear is that generally females achieve a higher BrAC than males when drinking the same amount of alcohol [14,57]. Similarly, other authors have found an association between alcohol blood concentration and visual deterioration, including Castro et al., who observed that a higher BrAC was associated with a greater deterioration of the VDI [15]. In contrast, other authors have reported that contrast sensitivity and stereopsis deterioration is not associated with the BAC level [5,22]; this seems reasonable, as not all visual variables are equally impaired by alcohol. For this reason, in studies evaluating various visual functions, it is appropriate to calculate a global visual index that includes all these visual variables. When using the BrAC level, the BMI, and the age as control variables, the effect of biological sex on the VDS was not significant, with the BrAC having a significant effect on the visual deterioration (VDS) as a function of biological sex. This indicates that the BrAC seems to be the main responsible of the differences in the VDS in males and females, due to a lower weight and the lower volume distribution of alcohol. However, the effect of the BMI, which was also correlated with the volume distribution of alcohol, was less important. Further information about these metabolic differences between males and females would be necessary to better understand the influence of these factors. Besides, for age as a covariate, no significant influence was observed, which is reasonable since there were no age differences between males and females in this study, as commented in the results. However, a limitation of this study is that our participants were between 20 and 30 years of age, so we cannot conclude that age has no effect on the visual deterioration caused by alcohol consumption, given that the analysis of this aspect is beyond the scope of this study.

## 5. Conclusions

Vision was strongly impaired after participants consumed a fixed moderate–high amount of alcohol (equal for all participants), such that they reached a breath alcohol concentration (BrAC) above the legal limit for driving in most countries (0.25 mg/L). Under dim lighting conditions, pupil size increased after alcohol consumption and the visual discrimination capacity was deteriorated, favoring halo perception. Monocular and binocular contrast sensitivity (CS) was negatively affected for all spatial frequencies, but in different ways, depending on the frequency being evaluated. Near and distance stereopsis were impaired under these conditions, particularly distance stereopsis. Finally, retinal image quality, assessed using the MTF cut-off, the Strehl Ratio, and the objective scatter index (OSI), was poorer after alcohol consumption for two artificial pupil sizes (of 4 and 5 mm). The deterioration of all these visual variables was greater in females, particularly the monocular CS, and the retinal image quality for a 5 mm pupil. The visual deterioration score (VDS), an overall score that includes the deterioration recorded for all the visual functions analyzed, was associated with the BrAC, which was higher in females. Likewise, the visual deterioration score (VDS) itself was higher for females, with significant main effects of BrAC being found, indicating that the higher visual deterioration observed in females was due to differences in the BrAC level reached and, to a lesser extent, to differences observed in the BMI. In light of these results, we propose the use of a visual deterioration score, similar to that studied here, as a reliable method for analyzing the visual deterioration that follows alcohol consumption, as well as under other conditions. Moreover, we believe that these findings should be considered when investigating the effects of alcohol intake on daily activities that require good visual performance.

## Figures and Tables

**Table 1:** Mean values of the parameters for the different visual functions analyzed under the two experimental conditions (baseline and after alcohol consumption, aAC): visual disturbance index (VDI), stereoacuity (near and distance), and contrast sensitivity (CS). Both VDI and CS monocular (MON) and binocular (BIN) values are given for the VDI and CS. Standard errors as well as statistical results and *p*-values are indicated.

	Baseline	aAC	Z; *p*-Value	Cohen’s d	Deterioration
VDI (MON)	0.21 ± 0.02	0.31 ± 0.03	Z(36) = −3.718 *p* < 0.001	1.545	−0.09 ± 0.02
VDI (BIN)	0.14 ± 0.01	0.19 ± 0.02	Z(36) = −2.826 *p* = 0.005	1.049	−0.05 ± 0.02
Pupil size (mm)	5.6 ± 0.2	5.9 ± 0.2	t(36) = −4.235 *p* < 0.001	0.696	−0.3 ± 0.1
Distance stereoacuity (arc sec)	52.7 ± 6.66	128.3 ± 14.14	Z(36) = −4.711 *p* < 0.001	2.449	−80.3 ± 13.92
Near stereoacuity (arc sec)	18.2 ± 2.76	38.6 ± 6.38	Z(36) = −4.482 *p* < 0.001	2.180	−20.0 ± 2.96
CS (MON)	124.9 ± 3.39	99.9 ± 3.62	t(36) = 6.690 *p* < 0.001	1.115	25.0 ± 3.62
CS (BIN)	149.8 ± 2.84	130.1 ± 4.76	t(36) = 5.906 *p* < 0.001	0.984	9.8 ± 1.61

**Table 2:** Mean values of the retinal image quality parameters (Strehl ratio and MTF cut-off for both artificial pupil sizes of 4 and 5 mm, and the OSI parameter) and the corresponding deterioration under the two experimental conditions (baseline and aAC). Standard errors and statistical parameters are also included.

Retinal Image Quality		Baseline	aAC	t; *p*-Value	Cohen’s d	Deterioration
Strehl ratio	4 mm	0.23 ± 0.01	0.20 ± 0.01	t(36) = 3.014; *p* = 0.005	0.191	0.03 ± 0.01
	5 mm	0.21 ± 0.01	0.17 ± 0.01	t(36) = 3.672; *p* < 0.001	1.211	0.03 ± 0.01
MTF cut-off (cpd)	4 mm	41.13 ±1.50	35.34 ± 1.58	t(36) = 5.492; *p* = 0.001	1.836	5.53 ± 0.92
	5 mm	36.31 ± 1.82	31.28 ± 1.65	t(36) = 4.328; *p* < 0.001	1.405	6.21 ± 1.47
OSI (4 mm)		0.56 ± 0.06	0.82 ± 0.10	Z(36) = −4.166 *p* < 0.001	1.880	−0.24 ± 0.06

**Table 3:** Results of the *t*-test for independent samples and Mann–Whitney U test (degrees of freedom = 35) showing how biological sex influences the deterioration of the different visual functions.

**Visual Variable**	**t Statistic**	***p*** **-Value**	**Cohen** **’** **s d**
Mon CS	−3.074	0.004 *	0.913
Bin CS	−1.510	0.140	0.489
Strehl ratio 4 mm	−1.037	0.307	0.356
Strehl ratio 5 mm	−2.217	0.034 *	0.710
MTF cut-off 4 mm	−1.566	0.127	0.527
MTF cut-off 5 mm	−2.189	0.036 *	0.702
**Visual Variable**	**Z Statistic**	***p*** **-Value**	**Cohen** **’** **s d**
Mon VDI	0.701	0.497	0.232
Bin VDI	0.838	0.407	0.278
Pupil size	1.472	0.177	0.457
OSI	1.418	0.158	0.568
Near stereoacuity	1.244	0.220	0.414
Distance Stereoacuity	0.901	0.390	0.294

Significant differences are indicated with an asterisk (*).

**Table 4:** Results of the ANCOVA test indicating the degrees of freedom, the F statistic, the significance level (*p*-value), and the effect size (ηp2) for the factor (*) and covariates (BrAC, BMI and age) included in the model.

Factor and Covariates	Degrees of Freedom	ANCOVA F	*p*-Value	Effect Size η_p_^2^
Fixed model	4	3.332	0.022	0.289
BrAC	1	4.535	0.041	0.124
Age	1	1.921	0.103	0.081
BMI	1	2.939	0.096	0.084
Biological sex *	1	4.246	0.290	0.035

## Data Availability

Available from the corresponding author on reasonable request.
